# Interaction of Ethylene with Ir_*n*_ (n = 1–10): From Bare Clusters to γ-Al_2_O_3_-Supported Nanoparticles

**DOI:** 10.3390/nano9030331

**Published:** 2019-03-02

**Authors:** Xue-Rong Shi, Yajing Zhang, Shibiao Zong, Wen Gu, Pan Ma, Na Lu

**Affiliations:** 1 School of Material Engineering, Shanghai University of Engineering Science, 333 Longteng Road, Songjiang District, Shanghai 201620, China; M050118113@sues.edu.cn (Y.Z.); M050117110@sues.edu.cn (S.Z.); guwen1-1@163.com (W.G.); mapan@sues.edu.cn (P.M.); 05160003@sues.edu.cn (N.L.); 2 Institute of Physical Chemistry, University of Innsbruck, Innrain 80-82, Innsbruck A-6020, Austria

**Keywords:** density functional theory (DFT), nucleation, metal clusters, alumina, ethylene

## Abstract

Comprehending the bond nature of ethylene-metal clusters at the atomic level is important for the design of nanocatalysts and their applications in the fields of fine chemistry and petroleum refining. The growth of Ir_*n*_ (n = 1–10) on γ–Al_2_O_3_(110) and ethylene adsorption on bare and γ–Al_2_O_3_(110)-supported Ir_*n*_ (n = 1–10) clusters were investigated using the density functional theory (DFT) approach. The mode stability of ethylene adsorption on the bare Ir_*n*_ clusters followed the order π > di-σ > B-T, with the exception of Ir_8_ where the π structure was less stable than the di-σ configuration. On supported Ir_*n*_ (n = 4–7 and 10) the stability sequence was π > di-σ > di-σ′ (at interface), while on supported Ir_*n*_ (n = 2, 3, 8, and 9) the sequence changed to di-σ > π > di-σ′ (at interface). Two-thirds of ethylene adsorption on the supported Ir_*n*_ clusters were weaker than its adsorption on the bare Ir_*n*_ clusters. The pre-adsorbed ethylene at the interface was found to facilitate the nucleation from the even-sized supported Ir_*n*_ to odd-sized Ir_*n*_ clusters, but hindered the nucleation from the odd-sized Ir_*n*_ to even-sized Ir_*n*_ clusters.

## 1. Introduction

Interaction of light alkenes with metal clusters has been widely studied in the past due to its important applications in the fields of fine chemistry and petroleum refining. As the smallest alkene and one of the most common probe molecules, the adsorption of ethylene on supported metal catalysts has been widely investigated [1,2,3,4,5]. Keppeler et al. [3] studied ethylene hydrogenation on the NaY and KL zeolite-supported Pt_13_ cluster and found ethane was the only product. 

Recently, many experimental works [6,7,8] have investigated the properties of nanosized metal clusters on alumina support. D’Ippolito et al. [8] found that the addition of iridium to SiO_2_–Al_2_O_3_ enhanced the decalin conversion in the selective ring opening reaction of decalin, while adding HCl barely affected the reaction. Argo et al. [9,10] found that iridium clusters (Ir_4_ and Ir_6_) on γ-Al_2_O_3_ rearranged slightly to adapt reactive intermediates in the reaction of ethylene hydrogenation, but remained intact using extended X-ray absorption fine structure (EXAFS) study. 

Many efforts have been carried out to investigate the adsorption and nucleation of transition metals on alumina using first-principles calculations [11]. Wang et al. [12] studied the growth of Ir_*n*_ (n = 1–5) clusters on the dehydrated and hydrated γ-Al_2_O_3_ surfaces. They found the surface hydroxyl hindered the adsorption but facilitated the nucleation of Ir_*n*_ clusters. Chen et al. [13] investigated the nucleation of Ir_*n*_ (n = 2–10) clusters on dehydrated γ-Al_2_O_3_(001) and MgO(100) surfaces using density functional theory approach. They found that the growth of Ir_*n*_ (n = 2–10) on these two surfaces was always exothermic. To our best knowledge, however, the study of ethylene interaction with bare Ir_*n*_ metal clusters and γ-Al_2_O_3_-supported Ir_*n*_ clusters is still unexplored.

The present work will focus on the effect of the support, which influences not only the adsorption performance of ethylene on Ir_*n*_, but also the structural features and cluster stability of the nanosized iridium. For this purpose, we use the DFT calculations to explore the growth of Ir_*n*_ (n = 1–10) clusters on γ-Al_2_O_3_ and discuss the interaction of ethylene with the bare and γ-Al_2_O_3_-supported Ir_*n*_ (n = 1–10) clusters. According to the previous work [14,15,16,17], the γ-Al_2_O_3_ surface probably is covered by the hydroxyl groups and the corresponding coverage of surface hydroxyl depends on the preparation temperature. In the reaction of ethylene hydrogenation, the γ-alumina-supported Ir catalysts are typically calcined at 598–673 K during the pretreatment process [9,10]. To correctly describe the surface structures under experimentally relevant conditions, we employ the surface structure constructed by Digne et al. [16] to perform our studies. 

## 2. Materials and Methods 

**Figure 1:**
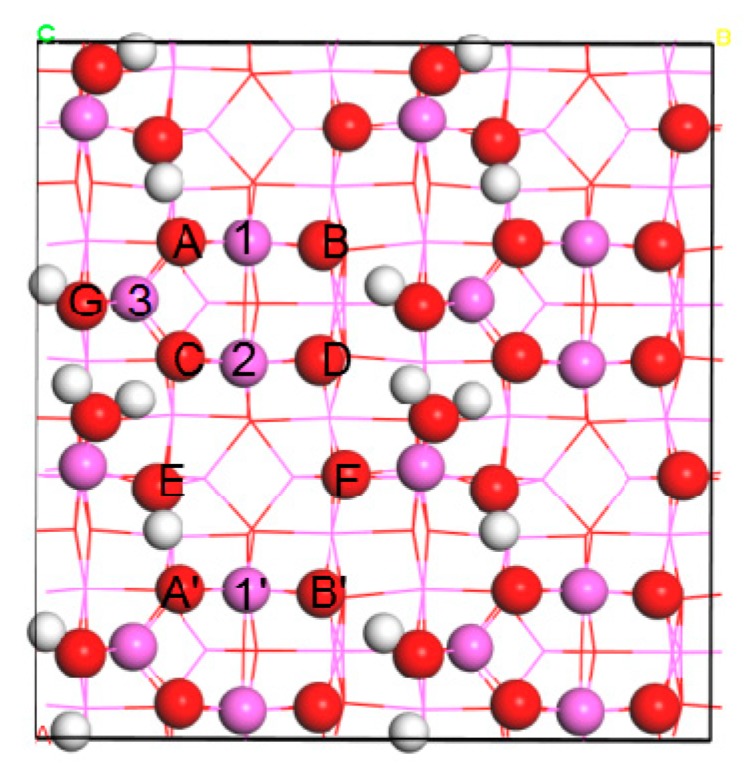
Top view of hydrated γ-Al_2_O_3_(110) surface with 5.9 OH/nm^2^. Atoms in the first layer and other layers are displayed in sphere and line forms, respectively. White: H, red: O, and pink: Al.

The hydrated γ-Al_2_O_3_(110) surface with a hydroxyl coverage of 5.9 OH/nm^2^ at 673 K was constructed according to [16]. As shown in Figure 1, the covering hydroxyl groups and the surface Al and O atoms on this hydrated (110) surface formed a distinct valley. To prevent the interaction between the neighboring Ir_*n*_ clusters, a large slab of 2 × 2 unit cell (surface area is ~272 Å^2^) with four layers of thickness containing 224 atoms was employed. Only the bottom two layers were fixed during the geometry optimizations. For the bare Ir_*n*_ systems, all atoms were relaxed.

We used the the Vienna ab initio simulation package (VASP) [18,19] to perform the spin-polarized DFT calculations. The Perdew–Wang exchange-correlation functional [20,21] and projector augmented wave (PAW) method [22] were employed with a cutoff energy of 400 eV. The force threshold of geometry optimization was set to 0.03 eV/Å. A gamma K-point for optimizations and 4 × 4 × 1 K-points for electronic structure calculations were used. All parameters in our calculations were carefully tested, with the change of the calculated E_ads_ smaller than 2%.

The adsorption energy E_ads_ of adsorbates species on substrate was calculated using

E_ads_ = E(Ads/Sub) − E(Sub) − E(Ads) (1)

E(Ads/Sub), E(Sub), and E(Ads) are the total energies of the energy minimized substrate with adsorbates, bare substrate, and gas-phase adsorbates, respectively.

The deformation energy of the adsorbate between its equilibrium structure in the gas phase and the adsorbed state was calculated by

E_def_(Ads) = E(Ads′) − E(Ads) (2)

Here E(Ads′) is the total energy of adsorbate species in the gas phase employing the structure displayed in the adsorbed state. Similarly, the deformation energy of the substrate E_def_(Sub) was calculated by

E_def_(Sub) = E(Sub′) − E(Sub) (3)

E(Sub′) is the total energy of the substrate retaining the adsorbed geometry, but with the adsorbates removed. The adsorbate–substrate interaction energy E_int_ was calculated by

E_int_ = E(Ads/Sub) − E(Ads′) − E(Sub′) (4)

For ethylene adsorption, the corresponding adsorption Gibbs free energy ∆G_ads_(T, P) was defined using

∆G_ads_(T, P) = E_ads_ − G^ɵ^(T) − RTln(P_C2H4_/P^ɵ^) (5)

G^ɵ^(T) contains the thermodynamical items of translation, vibration, and rotation of ethylene molecules in the gas phase. P_C2H4_ is the partial pressure of ethylene. We include zero-point vibrational energy in our present work. A detailed description of Equation (5) can be found in our previous work [23].

## 3. Results and Discussion

### 3.1. Gas-Phase Clusters

The structures of gas-phase Ir_*n*_ (n = 2–10) clusters, which are critical to comprehending the nucleation of Ir_*n*_ clusters on γ-Al_2_O_3_, have been well studied before [12,13]. Thus, although we considered different gas-phase Ir_*n*_ structures, only the most favorable geometries with the lowest energy are summarized in Table S1. The energetically preferred geometries for Ir_*n*_ (n = 3–8) were linear (D_∞h_), square planar (in D_4h_), square pyramid (C_4v_), triangular prism (D_3h_), side-face-capped triangular prism (C_2v_), and cubic structure (O_h_), respectively. The Ir_9_ cluster presented a C_s_ point group with one Ir atom bridged on two neighboring edge Ir atoms of the cube. The most stable Ir_10_ geometry yielded the configuration with the Ir_2_ dimer capping on one face of the cube. The Ir–Ir distance of the Ir_*n*_ (n = 2–10) clusters was in the range of 2.18–2.51 Å, which was shorter than the bulk Ir–Ir distance of 2.74 Å. A similar bond contraction was observed for the bare Rh clusters without ligands experimentally [24] and theoretically [25].

### 3.2. Small Ir_n_ Clusters on Hydrated γ-Al_2_O_3_(110)

**Figure 2:**
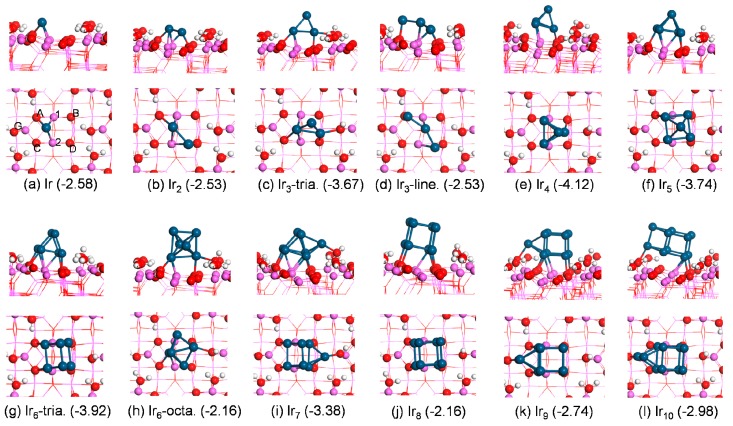
Side (top) and top views (bottom) of the most favorable Ir_*n*_ (*n* = 1–10) adsorption structures with the corresponding adsorption energies E_ads_ (in eV) on the hydrated *γ*-Al_2_O_3_(110) surface. Largest spheres: Ir; the other color/label scheme is identical to Figure 1.

For Ir adsorption, we considered a series of adsorption sites including seven top (O(A), O(B), O(C), O(D), O(F), Al(1), and Al(2)), ten bridge (O(A)-O(C), O(B)-O(D), O(D)-O(F), O(B′)-O(F), Al(1)-Al(2), Al(1′)-Al(2), O(A)-Al(1), O(B)-Al(1), O(C)-Al(2), and O(D)-Al(2)), and three hollow sites (O(A)-O(C)-Al(3), O(D)-O(F)-Al(2), and O(B′)-O(E′)-O(F)) (Figure 1). The most energetically favorable structures and corresponding adsorption energies E_ads_ are summarized in Figure 2 and Table S2, respectively. 

As shown in Figure 2, a single Ir atom preferred to bond to surface Al(1), Al(2), and O(A) atoms yielding E_ads_ of −2.58 eV. For Ir_2_ adsorption, both Ir atoms bonded to the surface, forming three Ir–Al bonds and two Ir–O bonds with E_ads_ of −2.53 eV. Two Ir atoms bonded to the same surface Al center. 

Unlike the case in the gas phase, the adsorbed Ir_3_ preferred the triangular configuration over the linear one. The adsorbed triangular structure presented E_ads_ of −3.67 eV with respect to the gas-phase triangular trimer, which was 1.14 eV lower in energy than the linear structure, while in the gas phase the former was 0.26 eV higher than the latter. The adsorbed triangular trimer presented a stand-up configuration with two Ir atoms binding to the surface and one Ir atom pointing to the air. While in the adsorbed linear trimer, each Ir atom bonded to the surface. 

For Ir_4_ adsorption, all attempts to obtain the square planar Ir_4_ cluster (the most stable configuration in gas phase) converted to the geometry with a bent rhombus on the surface. The reconstruction of small deposited metal clusters on the support was also observed for Pd_*n*_ clusters (n = 1–7) adsorption on α–Al_2_O_3_ (0001). Nigam and Majumder [26] found that the Pd_4_ deformed from the tetrahedral configuration in the gas phase into the bent rhombus in the adsorbed state on the Al_2_O_3_ surface. The most stable structure was a tetrahedron with three Ir atoms bonding to the surface and resulting in E_ads_ of −4.12 eV. Our results agree with the earlier experimental and theoretical reports. Using EXAFS spectroscopy, Argo et al. [9] found an Ir–Ir first-shell coordination number in γ-Al_2_O_3_-supported Ir_4_ nanoparticles of about 3. Our previous study showed that Rh_4_ also prefers the tetrahedral frame on γ-Al_2_O_3_ surfaces [25]. 

The adsorbed Ir_5_ retained the square pyramid geometry upon adsorption on the surface, with four Ir atoms bonding to the substrate and yielding E_ads_ of −3.74 eV.

Analogous to the case in the gas phase, the most energetically preferred Ir_6_ geometry was a triangular prism with E_ads_ of −3.92 eV. In the adsorbed state, four Ir atoms made contact with the substrate and two Ir atoms kept away from the surface. The most stable octahedral structure of adsorbed Ir_6_ was less stable by 1.76 eV (higher in energy) than the triangular prism on the γ-Al_2_O_3_ support, and in the gas phase the energy difference between each was 0.70 eV. The coordinates RMSD (root-mean-square deviation) between the triangular prism configuration and the octahedral structure for the Ir_6_ cluster were ~0.94 in the gas phase and ~0.98 for the supported case, respectively, which is consistent with the energy difference between the two. The octahedral adsorbed Ir_6_ cluster bonded to the substrate via two Ir atoms (Figure 2h). This was inconsistent with the experimental observation [9,26,27], but in agreement with the previous calculations [13,28,29]. Experimentally, the octahedral Ir_6_ commonly exists in the form of Ir_6_ complexes [9]. Argo et al. [9] used the octahedral frame of [Ir_6_(CO)_15_]^2−^ upon its decarbonylation to obtain an Ir_6_ cluster on the γ-Al_2_O_3_ support and found that Ir_6_ maintained the octahedral frame after decarbonylation using EXAFS measurements. Here, the CO ligands helped it to keep the octahedral frame, while several theoretical studies [13,28,29] found that the bare gas-phase Ir_6_ cluster without any ligand favored a triangular prism with D_3h_ symmetry over the O_h_ octahedral structure.

For Ir_7_ adsorption, the energetically preferred structure yielded a similar configuration to the gas-phase cluster with E_ads_ of −3.38 eV. The additional Ir atom to the adsorbed triangular prism Ir_6_ cluster bonded to the oxygen atom in a surface hydroxyl. A surface distortion (Figure 2i) was observed upon the adsorption of the Ir_7_ cluster, where the bonded hydroxyl moved upwards to make contact with the Ir atom. The similar adsorbate-induced support rearrangement was found for triangular Ir_3_ adsorption on γ-Al_2_O_3_ (Figure 2c). 

The most stable configuration for Ir_8_ adsorption on alumina was a cubic Ir_8_ cluster with four Ir atoms bonding to the surface and resulting in E_ads_ of −2.16 eV (Figure 2j). The most favorable structures for Ir_9_ and Ir_10_ adsorption exhibited E_ads_ of −2.74 and −2.98 eV, respectively. As shown in Figure 2k,l, one Ir atom in the adsorbed Ir_9_ and Ir_10_ cluster bonded to the oxygen center in a surface hydroxyl group. 

Similar to our previous finding for Rh adsorption on the hydrated γ-Al_2_O_3_(110) surface [25], Ir_*n*_ clusters preferred to adsorb in the valley of the surface O and Al sites instead of on the covering hydroxyls layer, indicating the nanosized metal cluster can retard the transformation of γ-Al_2_O_3_ to AlOOH by pre-adsorption on the transformation site. This is consistent with the previous experimental [30] and theoretical [25] observations, confirming that it may be a general effect for a large number of nanosized transition metals. 

In summary, Ir_*n*_ preferred to adsorb on the no-hydroxyls-covered area. When cluster diameter (to simplify, the cluster diameter is defined by the longest distance of M–M in the cluster) was smaller than 4.11 Å (e.g., n = 1–6 and 8), the Ir_*n*_ cluster bonded to the surface O and Al atoms only. When cluster diameter was larger than 4.21 Å (n = 7, 9, and 10), besides bonding to the surface O and Al atoms, the Ir_*n*_ cluster bonded to the oxygen of surface hydroxyl as well. The γ–Al_2_O_3_ (110) support changed the morphological features and the cluster stability of Ir_3_ and Ir_4_ upon their adsorption on the support. The Ir–Ir distance of adsorbed Ir_*n*_ (n = 2–10) in the basal plane underwent an elongation compared with the gas-phase Ir_*n*_ clusters. 

### 3.3. Adsorption of Ethylene on Bare Ir_n_

For ethylene adsorption on the bare Ir_*n*_ cluster, three adsorption modes were considered: di-σ mode, with two carbon ends of ethylene binding to two substrate atoms; π mode, with two carbon ends of ethylene binding to one substrate atom; and a bridge-top (B-T) mode, with one carbon bridging two substrate atoms and the other carbon binding to one substrate atom.

**Figure 3:**
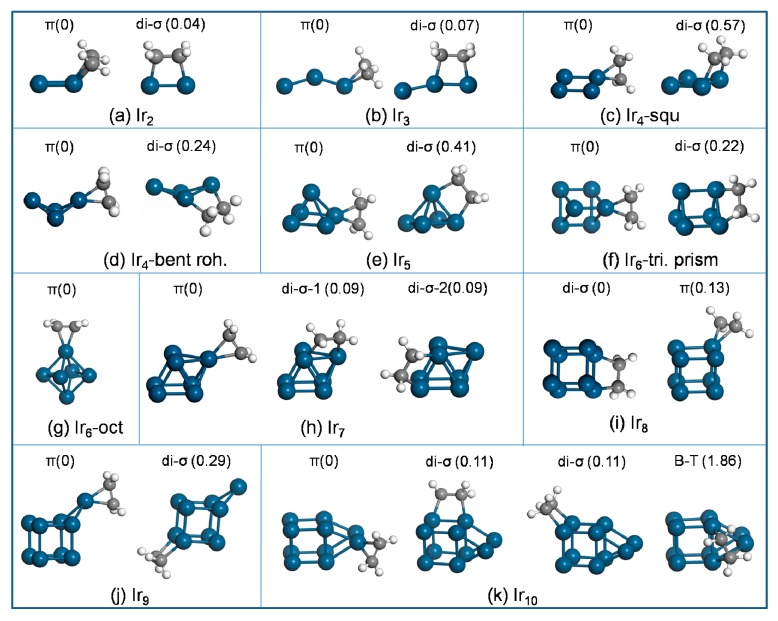
The most stable structure for each ethylene-binding mode on bare Ir_*n*_. The energy difference (eV) with respect to the most stable configuration (energy set to 0) is labeled at the top of the figure.

Ethylene adsorption on atomic Ir yielded the largest E_ads_ of −2.96 eV, suggesting the strongest ethylene binding of all considered Ir_*n*_ (n = 1–10) clusters (Table 1). Ethylene adsorption on the Ir_2_ cluster (Figure 3a) via π-bound mode yielded an adsorption energy E_ads_ of −1.79 eV, which was very close to the di-σ-bound mode that featured E_ads_ of −1.75 eV. This suggests very similar adsorbate binding for both modes at 0 K.

Geometry optimization of ethylene adsorption on the bare Ir_3_ cluster yielded three different local-energy minima, and those with the lowest total energy for each mode are shown in Figure 3b. The E_ads_, −1.82 eV (π-bound mode) and −1.75 eV (di-σ-bound mode), differ only slightly. The adsorption of ethylene induced a strong deformation of the Ir_3_ cluster, where Ir_3_ changed from the linear structure in the free phase to the bent configuration with the adsorbate. The deformation energy E_def_(Ir_3_) reached as high as 0.23 (0.15) eV for the π (di-σ)-bound mode.

For ethylene adsorption on the square Ir_4_ cluster (Figure 3c), the calculations found two local equilibrium geometries for each mode. The π-bound state was more favorable than the di-σ-bound mode, where E_ads_ of the π-bound mode was −2.46 eV and the di-σ-bound state yielded E_ads_ of −1.89 eV. To compare with the supported Ir_4_ cluster, ethylene adsorption on the bent-rhombus Ir_4_ cluster was studied as well. Ethylene adsorption yielded four di-σ and two π local minima, and the energetically preferred structure for each mode is provided in Figure 3d. The di-σ mode was less stable than the π mode by 0.24 eV (higher in energy).

Ethylene adsorption on Ir_5_ (two local equilibrium geometries for each mode are found) preferred the π-bound state to the di-σ-bound mode, which accounts for E_ads_ of −2.30 eV (π) and −1.80 eV (di-σ). 

Ethylene stabilizes on the triangular-prism Ir_6_ cluster, and the most favorable adsorption geometries are shown in Figure 3f. Ethylene adsorption on the triangular-prism Ir_6_ preferred the π state to the di-σ mode. The corresponding adsorption energies were −2.06 and −1.84 eV for the π-bound state and di-σ-bound mode, respectively. Similarly, ethylene adsorption on the octahedral Ir_6_ cluster (Figure 3g) via the π-bound state was more stable than it was via the di-σ-bound mode, with an energy difference of 0.46 eV.

Geometry optimization of ethylene adsorption on the Ir_7_ cluster yielded three π and four di-σ local-energy minima. The most stable adsorption geometries of ethylene on the Ir_7_ cluster for each mode are sketched in Figure 3h. Our results show that the π structure yields E_ads_ of −1.94 eV, which is more favorable than the di-σ structure by 0.09 eV (lower in energy).

The energetically preferred adsorption geometries of ethylene on the Ir_8_ cluster are sketched in Figure 3i. The calculations yield E_ads_ of −1.63 eV (π) and −1.76 eV (di-σ), suggesting stronger binding for the di-σ structure compared with the π state.

For ethylene adsorption on Ir_9_, the calculations found three π and five di-σ local-energy minima structures, and the most stable one is shown in Figure 3j. The most stable π and di-σ structures yielded E_ads_ of −2.11 and −1.82 eV, respectively, indicating a stronger stability of the π mode.

The most stable adsorption geometry of ethylene on the bare Ir_10_ cluster via π-bound mode (of three local equilibrium geometries found) is sketched in Figure 3k. The stability of ethylene adsorption decreased in the sequence of π > di-σ > B-T. Among the obtained three local equilibrium geometries, the most stable π structure yielded E_ads_ of −1.88 eV, which was 0.11 eV lower than the most stable di-σ structure (of seven local equilibrium geometries). Two di-σ structures yielded identical E_ads_ of −1.77 eV. The B-T configuration (Figure 3k) was the least stable, with much smaller E_ads_ of −0.02 eV. Note that the B-T structure was only available on Ir_10_. All attempts to obtain the B-T configuration on the other Ir_*n*_ clusters resulted in either the π structure or the di-σ mode.

In summary, the stability of ethylene adsorption on the bare Ir_*n*_ clusters decreased in the sequence of π > di-σ > B-T with one exception of Ir_8_ where the di-σ structure was energetically preferred over the π structure. 

To analyze the adsorption energy in more details, we divided it into three contributions according to E_ads_ = E_def_(C_2_H_4_) + E_def_(Ir_*n*_) + E_int_. From the data summarized in Table 1, we can see that the ethylene deformation energies for the di-σ and π structures were within the energy range of 1.53–1.85 and 0.45–0.53 eV, respectively. The deformation energy of ethylene accompanied the adsorption of ethylene along with the C−C bond elongation. The di-σ structure always induced a greater elongation regarding the gas phase than the π mode in our studies, which is in agreement with previous theoretical studies [31,32]. The C−C bond distance in the di-σ mode enlarged to ~1.51Å from 1.33 Å in the gas phase, while the π mode caused a smaller C−C bond extension (~1.43 Å).

Further analysis shows that the deformation of the adsorbate was much stronger than that of the substrate. As the deformation energies of the adsorbed ethylene were larger than 0.45 eV, the deformation energies of Ir_*n*_ clusters were rather small, below 0.23 eV. The energy cost for the deformation could have been compensated by the interaction energy between the adsorbates and the substrate. Despite the cluster–ethylene interaction energy in the di-σ mode always being larger than that in the π mode, it could not compensate for the energy cost difference of the deformation between two modes on most of the Ir_*n*_ clusters (excluding Ir_8_). As a result, ethylene preferred to adsorb on the bare clusters via the π mode, except for the Ir_8_ cluster. The reverse preference of adsorption mode on Ir_8_ was the same with Ir(111) [33], where the di-σ mode was more favorable than the π mode.

### 3.4. Adsorption of Ethylene on Al_2_O_3_(110)-Supported Ir_n_

**Figure 4:**
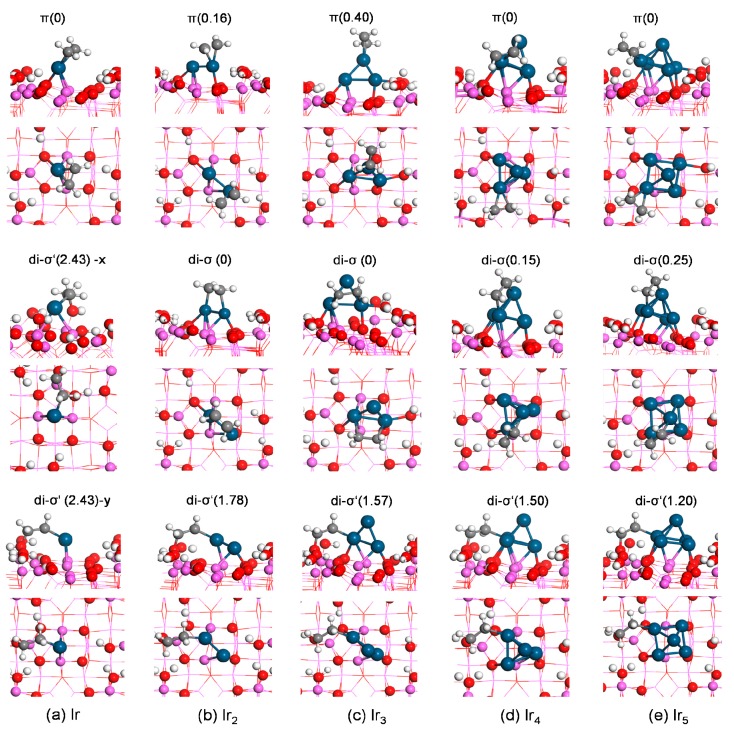
Side view (top) and top view (bottom) of most stable structure for each ethylene-binding mode on γ-Al_2_O_3_(110)-supported Ir_*n*_ (n = 1–5). The energy difference (eV) with respect to the most stable configuration (energy set to 0) is labeled at the top of the structure. For the di-σ′ mode at the interface on supported Ir monomer, x and y are the different views of the same structure along two directions. The largest spheres are Ir, and the other color/label scheme is identical to Figure 1.

**Figure 5:**
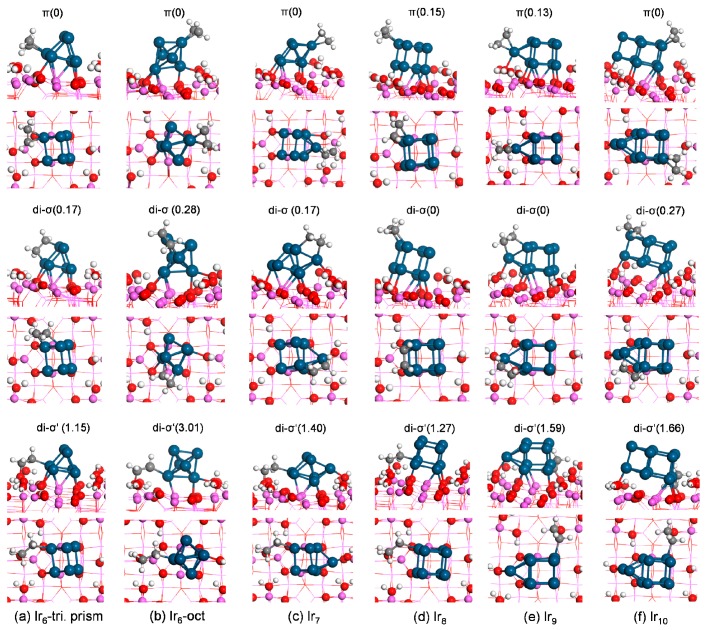
Side (top row) and top views (bottom row) of the most stable structure for each ethylene-binding mode on γ-Al_2_O_3_(110)-supported Ir_*n*_ (n = 6–10). The energy difference (eV) with respect to the most stable configuration (energy set to 0) is labeled at the top of the figure. The color/label scheme is identical to Figure 4.

Next, we studied the adsorption of ethylene on hydrated γ-Al_2_O_3_(110)-supported Ir_*n*_ clusters. For ethylene adsorption on Ir_*n*_/γ-Al_2_O_3_, besides the three scenarios described on the bare Ir_*n*_ cluster, one more scenario was considered: the di-σ′ mode at the interface with one carbon atom on the Ir_*n*_ cluster and one carbon atom on the γ-Al_2_O_3_ support. Therefore, for ethylene adsorption on Ir_*n*_/γ-Al_2_O_3_, we considered four possible adsorption geometries, including three modes on the supported Ir_*n*_ cluster (π, B-T, and di-σ) and one mode at the interface (di-σ′). The most stable configuration for each mode and their corresponding energies are summarized in Figure 4 and Figure 5 and Table 2.

Ethylene adsorption yielded almost the identical adsorption energy of −2.95 eV both on Ir_1_/γ-Al_2_O_3_(110) and on atomic Ir. The di-σ′ mode at the interface, where one carbon atom of ethylene bonds to the Ir atom and the other carbon end bonds to the oxygen site of the surface hydroxyl group, was less stable by 2.42 eV in energy. 

For ethylene adsorption on Ir_2_/γ-Al_2_O_3_(110), the di-σ structure was energetically more favorable than the π state (Figure 4b). The adsorption energy of the di-σ mode was −2.26 eV, while the π mode gave E_ads_ of −2.10 eV. As was the case for Ir_1_/γ-Al_2_O_3_(110), the di-σ′ structure at the interface resulted in much higher E_ads_ of −0.48 eV, indicating this structure was less favorable than the di-σ and π structures on the supported Ir_2_ cluster thermodynamically.

Three π and three di-σ structures were obtained for ethylene adsorption on Ir_3_/γ-Al_2_O_3_(110) through geometry optimization. In the most stable π mode (Figure 4c), ethylene preferred to adsorb on the upper Ir site while in the most favorable di-σ mode, two carbon ends bonded to the bottom Ir centers. The adsorption energies of the di-σ structure and the π mode were −1.99 and −1.75 eV, respectively, indicating the preference of the di-σ structure. The most stable di-σ′ structure at the interface (of three obtained structures) yielded E_ads_ of −0.58 eV.

Our calculations obtained five di-σ, six π, three monodentate (M) with one carbon end adsorbing on a metal site (Figure S1a), and two di-σ′ structures at the interface for ethylene adsorption on Ir_4_/γ-Al_2_O_3_. The most stable π mode yielded E_ads_ of −1.94 eV. While the most stable di-σ structure yielded E_ads_ of −1.79 eV (Figure 4d). The di-σ′ structure at the interface with one carbon end at a bottom Ir site and one carbon end at the oxygen O(G) site in the hydroxyl group of the γ-Al_2_O_3_(110) surface yielded E_ads_ of −0.44 eV. The most stable M mode yielded E_ads_ of –0.66 eV. The stability of the ethylene adsorption mode decreased in the order of π > di-σ > M > di-σ′ (at interface). 

For ethylene adsorption on Ir_5_/γ-Al_2_O_3_, ethylene binded to one bottom Ir atom resulting in E_ads_ of −1.84 eV in the most stable π mode (of four local equilibrium geometries found). Ethylene adsorbed on the substrate through its two C atoms bonding to one bottom Ir and one upper Ir (di-σ) yielding E_ads_ of −1.59 eV. Two di-σ′ structures at the interface yielded very close E_ads_, with an energy difference of 0.08 eV (the more stable one is shown in Figure 4e).

On Ir_6_/γ-Al_2_O_3_, the most stable π-bound ethylene yielded E_ads_ of −1.71 eV (Figure 5a). It adsorbed on a bottom Ir atom. The most stable di-σ-bound structure yielded an adsorption energy of −1.54 eV and bridged one top and one bottom Ir atoms. The most stable di-σ′ structure at the interface (of two local equilibrium geometries) on Ir_6_/γ-Al_2_O_3_(110), with one carbon atom on a bottom Ir atom and one carbon atom on the oxygen site of surface hydroxyl, yielded E_ads_ of −0.56 eV.

Although the most stable supported Ir_6_ exhibited a triangular-prism configuration and the octahedral structure was less stable by 1.76 eV (higher in energy), as discussed in Section 3.2, the supported octahedral Ir_6_ cluster was observed by the experiments. Therefore, the adsorption of ethylene on the most stable supported octahedral Ir_6_ cluster was studied for comparison. On Ir_6oct_/γ-Al_2_O_3_, the most stable π-bound ethylene yielded E_ads_ of −2.19 eV (Figure 5b). It adsorbed on a top Ir atom with two Ir–C bond lengths both of 2.11 Å. The most stable di-σ-bound structure yielded an adsorption energy of −1.91 eV. It bridged one top Ir atom and one middle Ir atom. The present results agree with the previous experimental observation [9] and theoretical results [34,35]. Qi et al. [34] and Valero et al. [35] discovered that the π adsorption mode was more stable than the di-σ mode for ethylene adsorption on Ir_4-C_/γ-Al_2_O_3_(110) and Pd_4_/γ-Al_2_O_3_(110) catalyst. Argo et al. [9] observed that the π adsorption mode was directly relevant to the hydrogenation reaction on Ir_*n*_/γ-Al_2_O_3_ (n = 4 and 6). The most stable M structure (of three local-energy minimum structures, Figure S1b) yielded E_ads_ of −0.62 eV. The di-σ′ (at interface) structure yielded E_ads_ of +0.82 eV suggesting the adsorption was meta-stable and strongly endothermic.

For ethylene adsorption on Ir_7_/γ-Al_2_O_3_, five local equilibrium geometries for both the π and di-σ modes were found. The most stable π state resulted in E_ads_ of −1.96 eV (Figure 5c). The most stable di-σ structure was less stable than the π state, with E_ads_ of −1.79 eV. The di-σ′ structure at the interface yielded E_ads_ of −0.56 eV. Therefore, the stability of these structures decreases in the order π > di-σ > di-σ′ (at interface).

We found three π and four di-σ-bound local-energy minimum structures for ethylene adsorption on Ir_8_/γ-Al_2_O_3_. Analogous to the case on the bare Ir_8_ cluster, the di-σ configuration was energetically preferred over the π state by 0.18 eV (lower in energy). For the most stable π and di-σ structures, ethylene preferred to adsorb on the upper Ir atoms away from the interface (Figure 5d). The most stable di-σ′ structure at the interface among two obtained local minima yielded an adsorption energy of −0.25 eV, suggesting less stability than the other two modes. 

For ethylene adsorption on Ir_9_/γ-Al_2_O_3_, geometry optimization obtained four π and three di-σ structures. In the most stable π and di-σ structures, ethylene preferred to adsorb on the Ir atom, capping on the face of the cube (Figure 5e). Unlike the case on the bare Ir_9_ cluster, the π structure was less stable than the di-σ state. The corresponding adsorption energies E_ads_ were −1.61 and −1.74 eV for the π and di-σ structures, respectively. For ethylene adsorption at the interface, the adsorption was nearly neutral, with E_ads_ of −0.02 eV.

For ethylene adsorption on Ir_10_/γ-Al_2_O_3_, the most stable π (of five local minima) and di-σ (of four local equilibrium geometries) structures are provided in Figure 5f. Ethylene adsorbed on the upper Ir atom of the cube in the most stable π mode. In the most stable di-σ mode, ethylene adsorbed on the top surface away from the interface where it bridged one Ir atom of the cube and one caped Ir atom. The most stable π structure yielded an adsorption energy of −1.59 eV, 0.27 eV lower in energy than the most stable di-σ state and suggesting the preference of π structure. Similar to the case on supported Ir_9_, ethylene adsorption at the interface of Ir_10_/γ-Al_2_O_3_, with one carbon bonding to one surface oxygen atom and the other carbon bonding to one bottom Ir site, was slightly endothermic, with E_ads_ of +0.07 eV.

As shown in Table 1 and Table 2, the di-σ mode (either at the interface or on the Ir_*n*_ cluster) caused a stronger distortion of the adsorbed ethylene associated with the larger deformation energy E_def_(C_2_H_4_) than the π state. This was due to the fundamental nature of the bonds. The bonding in these modes involved a rehybridization of the carbon centers. In Table 1 and Table 2, we provide the mean hybridization value according to the work of [35]. According to this work, the mean hybridization value of the gas-phase ethylene—whose carbon center exhibits *sp^2^* hybridization—was 2, and the carbon center with *sp^3^* hybridization (e.g., C in ethane) yielded the mean hybridization value of 3. We found the mean hybridization value of the di-σ′ mode at the interface was about 3, indicating a complete rehybridization of the carbon centers from *sp^2^* in the gas phase to *sp^3^* in the adsorbed state. The di-σ structure at the interface exhibited the same structure with the gas-phase ethane where two H atoms of ethane were substituted by one Ir atom and one surface O (see Figure 3a). The mean hybridization values of the di-σ and π states on the Ir_*n*_ cluster were ~2.45 and ~2.85, indicating a weaker rehybridization of the carbon centers in the π state than the di-σ structures regarding the gas-phase ethylene (the mean hybridization value was 2). These results reveal an electron transfer from the support to π* orbitals of ethylene. A linear relationship between the deformation energy of ethylene and the mean hybridization value of the carbon centers can be observed in Figure S2.

It is noted that Ir_6oct/_*γ*-Al_2_O_3_ exhibited the largest deformation energy upon ethylene adsorption, suggesting the strongest reconstruction among all considered Ir_*n*_/*γ*-Al_2_O_3_. Because of the steric hindrance effect, which limits space at the interface, the supported octahedral Ir_6_ rearranged itself to accommodate the adsorbed ethylene molecule. Meanwhile the ethylene-support interaction energy was not large enough to balance the deformation energies, resulting in the largest positive E_ads_ of +0.82 eV and indicating the adsorption at the interface was the weakest among all the considered configurations and strongly meta-stable.

### 3.5. Thermodynamics

**Figure 6:**
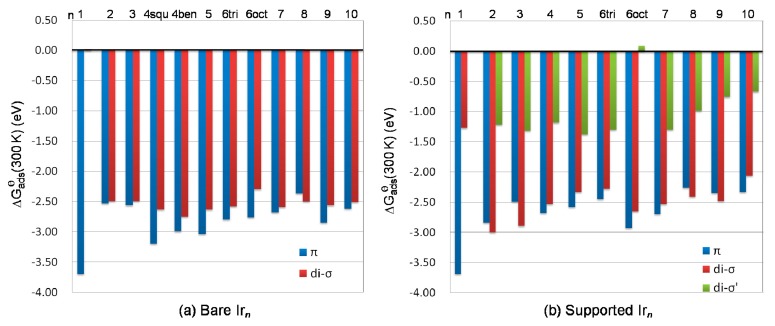
Standard adsorption Gibbs free energy at 300 K; ∆G^q^_ads_(300 K) of ethylene adsorption on (a) bare Ir_*n*_ clusters and (b) hydrated (110) *γ*-Al_2_O_3_-supported Ir_*n*_ clusters.

The adsorption Gibbs free energy ∆G_ads_(T, P) is shown in Figure 6. According to the previous work [36,37,38,39], typical molecular adsorption enthalpies for ethylene on silica-supported metal (Pt and Pd) surfaces are about 1.20–1.40 eV in absolute size at 300 K. Our calculation showed that standard adsorption Gibbs free energy at 300 K, with the partial pressure of ethylene at 1 atm, ∆G^ɵ^_ads_(300 K) for ethylene adsorption on the Ir(111) surface [33], fell within the same thermodynamic window as ∆G^ɵ^_ads_(300 K) of 1.32–1.45 eV in absolute size at 1/3 monolayer coverage. Meanwhile, for ethylene adsorption on *γ*-Al_2_O_3_-supported Ir_*n*_ clusters, the calculated ∆G^ɵ^_ads_(300 K) for the most stable π and di-σ structures yielded an energy range between −2.07 and −3.70 eV (Figure 6). This suggest that the ethylene–Ir_*n*_ interactions were much stronger than the ethylene–Ir(111) interactions. And from the thermodynamic view, ethylene adsorption on the bare and supported Ir_*n*_ clusters were much more favorable than those on the Ir(111) surface. 

The di-σ′ structure at the interface gave an energy range between +0.09 and −1.38 eV. Due to the limited space at the interface for the supported octahedral Ir_6oct_ cluster, ethylene adsorption at the interface via di-σ′ yielded a positive ∆G^ɵ^_ads_(300 K) value, indicating ethylene adsorption at the interface at 300 K with partial pressure of ethylene of 1 atm is thermodynamically unfavorable.

### 3.6. Analysis of Electronic Properties 

To know more about the charge redistribution upon the adsorption, we examined the local charge flow for adsorbed monomer, Ir_4_, and Ir_10_ systems using electron density difference maps. The electron density difference (∆*ρ*) was calculated by

∆*ρ* = *ρ*(Ads/Sub) − *ρ*(Ads)_fix_ − *ρ*(Sub)_fix_ (6)

where *ρ*(Ads/Sub) is the total electron density of the adsorbates/substrate complex, *ρ*(Ads)_fix_, and *ρ*(Sub)_fix_ are the electron densities of the isolated adsorbates and substrate in the same geometry as the adsorbed state, respectively. 

**Figure 7:**
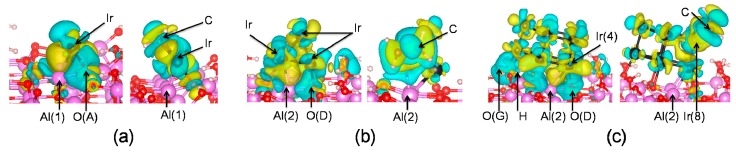
Electron density difference map for hydrated γ-Al_2_O_3_(110)-supported (**a**) Ir atom, (**b**) Ir_4_, and (**c**) Ir_10_ systems. Left column: Ir_*n*_ cluster adsorbed on γ-Al_2_O_3_; right column: ethylene adsorbed on Ir_*n*_/γ-Al_2_O_3_. Depletion regions: blue; accumulation region: yellow.

In the electron density difference maps (Figure 7), some *d* orbitals of Ir were depleted upon Ir_*n*_ cluster adsorption on the surface, which was associated with the charge redistribution of the formed Ir–O and Ir–Al bonds. Oxygen atoms, which bond to Ir atoms, lose electrons during the adsorption process, causing decreased electron density along the Ir–O bond, while in the regions of the Ir–Al bond, electron density increases. Similar phenomena have been observed for Pd [40] and Rh [25] cluster adsorption on *γ*-Al_2_O_3_. The depletion of Rh (Pd) *d* orbitals during its adsorption on *γ*-Al_2_O_3_ was balanced by increased electron density along the Rh(Pd)–Al bond. 

As shown in Figure 7, electrons previously accumulated along Ir–Al bonds in Ir_*n*_/γ-Al_2_O_3_ were transferred to the Ir_*n*_/C_2_H_4_ part upon C_2_H_4_ adsorption on γ-Al_2_O_3_-supported Ir_*n*_. This suggests that the adsorption of ethylene on γ-Al_2_O_3_-supported Ir_*n*_ influences the charge distribution at the metal–alumina interface. The following projected density of states (PDOS) analysis further confirms this statement.

**Figure 8:**
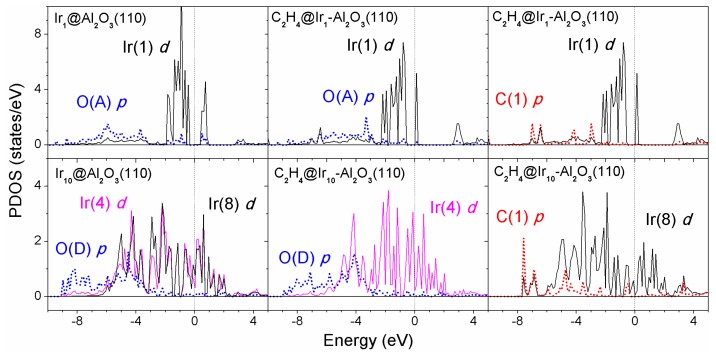
Density of states for the supported Ir_1_ (upper row) and Ir_10_ (bottom row) clusters on the hydrated *γ*-Al_2_O_3_ (110) surface projected on the bonded Ir atom, surface O, and C in the adsorbed ethylene (π mode) before and after ethylene adsorption. (Dotted lines: p states; solid lines: d states.).

The PDOS are summarized in Figure 8 for the Ir_1_/*γ*-Al_2_O_3_ and Ir_10_/*γ*-Al_2_O_3_ systems before and after ethylene adsorption. O(A)-Ir(1) and O(D)-Ir(4) were chosen to analyze the metal–support interaction for Ir_1_/*γ*-Al_2_O_3_ and Ir_10_/*γ*-Al_2_O_3_, respectively. 

Before ethylene adsorption, the *d* band states of Ir monomer on the alumina support were quite localized and showed an energy gap of ~0.8 eV, while for the supported Ir_10_ cluster, broad and delocalized *d* states (either Ir(4) or Ir(8) atoms) showed up and exhibited a finite density of states at the Fermi level. After ethylene adsorption, the *d* band states became a little smoother for the supported Ir monomer but still localized at the metal cluster, while for the supported Ir_10_ cluster the *d* band states became sharper upon ethylene adsorption. We note that the Ir atom in contact with the support but away from the adsorbed ethylene—Ir(4) as labeled—yielded sharper *d* band states after ethylene adsorption compared with the case before ethylene adsorption. This means the adsorbed ethylene induced charge redistribution at the iridium–alumina interface. The *p* orbitals of oxygen (carbon) atoms strongly mixed with the low-energy (typically below −3 eV) *d* states of Ir atoms. 

### 3.7. Effect of Adsorbed Ethylene on Nucleation of Ir_n_ Clusters on γ-Al_2_O_3_

We notice that the di-σ′ adsorption mode at the interface of Ir_*n*_(*n* = 1–8)/*γ*-Al_2_O_3_ occurred at the same place. This raises the question of whether the pre-adsorbed ethylene at the interface would have affected the growth of Ir_*n*_ clusters on the support. To answer this question, we calculated the nucleation energy according to the following equations. 

The nucleation or growth energy of Ir_*n*_ clusters from a combination of an adsorbed monomer and an Ir_*n*−1_ was defined by

E_nuc_ = E(Ir_*n*_/*γ*-Al_2_O_3_) + E(*γ*-Al_2_O_3_) − E(Ir_*n*−1_/*γ*-Al_2_O_3_) − E(Ir_1_/*γ*-Al_2_O_3_) (7)

For pre-adsorbed ethylene at the interface (di-σ′ mode) of Ir_*n*_/*γ*-Al_2_O_3_, the nucleation energy E_nuc_ was obtained by

E_nuc_ = E(C_2_H_4_-Ir_*n*_/*γ*-Al_2_O_3_) + E(*γ*-Al_2_O_3_) − E(C_2_H_4_-Ir_*n*−1_/*γ*-Al_2_O_3_) − E(Ir_1_/*γ*-Al_2_O_3_) (8)

For the nucleation of the gas-phase Ir_*n*_ clusters, the nucleation energy E_nuc_ was calculated using

E_nuc_ = E(Ir_*n*_) − E(Ir_*n*−1_) − E(Ir_1_) (9)

For pre-adsorbed ethylene on the gas-phase Ir_*n*_ clusters, the nucleation energy was defined by

E_nuc_ = E(C_2_H_4_/Ir_*n*_) − E(C_2_H_4_-Ir_*n*−1_) − E(Ir_1_) (10)

Only the most favorable structure is considered for each cluster size.

**Figure 9:**
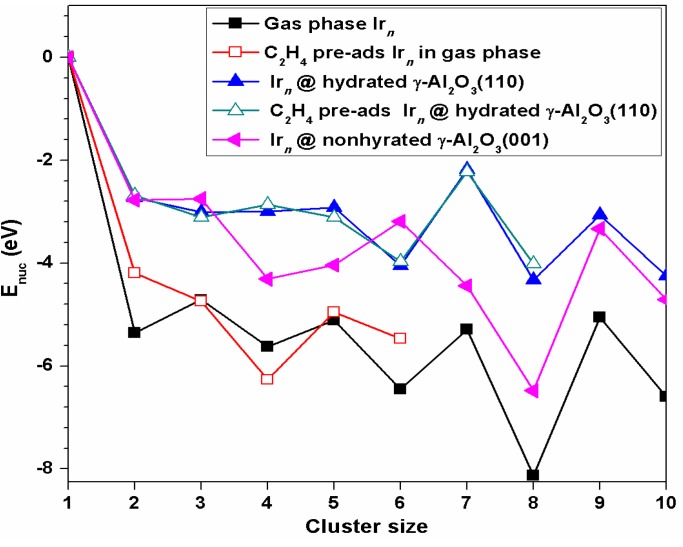
Nucleation energies E_nuc_ of the gas-phase Ir_*n*_ cluster; ethylene pre-adsorbed Ir_*n*_ in the phase via the π mode; Ir_*n*_ on hydrated *γ*-Al_2_O_3_(110); and ethylene pre-adsorbed at the interface of Ir_*n*_/*γ*-Al_2_O_3_ (110). For comparison, nucleation energy for Ir_*n*_ on *γ*-Al_2_O_3_(001) [13] is also included.

As shown in Figure 9, the nucleation energies for all Ir cluster sizes we considered were negative, indicating the critical cluster size for Ir growth is 2. Each nucleation step was exothermic, indicating the nucleation process is thermodynamically favorable. In other words, Ir atoms prefer to grow into nanosize Ir_*n*_ clusters atom by atom both in the gas phase and on the *γ*-Al_2_O_3_ surface, which is consistent with the experimental observation. Yentekakis et al. [41] observed the Ir particle agglomeration on *γ*-Al_2_O_3_ in the methane dry reformation reaction using transmission electron microscopy. 

An E_nuc_ comparison between hydrated (110) and non-hydrated (001) *γ*-Al_2_O_3_ surfaces revealed that except for Ir_3_ and Ir_6_ clusters, the nucleation of Ir_*n*_ clusters were less favorable on the hydrated *γ*-Al_2_O_3_ (110) than the non-hydrated *γ*-Al_2_O_3_ (001). For Ir_3_ and Ir_6_ clusters, the trend was reversed. The corresponding E_nuc_ for Ir_3_ and Ir_6_ on the hydrated (110) surface was 0.26 and 0.86 eV lower than those on the non-hydrated (001) surface, respectively. It should be pointed out that Ir_6_ exhibited different adsorption configurations on two surfaces. Chen et al. [13] found the most stable Ir_6_ adsorption configuration was an octahedron structure on the non-hydrated *γ*-Al_2_O_3_(001) surface, while on the hydrated *γ*-Al_2_O_3_(110) we found the octahedron structure of Ir_6_ was less stable by 1.76 eV (higher in energy) than the triangular prism (which was the most stable in the gas phase also).

A comparison of the nucleation energies of Ir_*n*_ on the hydrated *γ*-Al_2_O_3_(110) and pre-adsorbed ethylene at the interface (di-σ′ mode) of Ir_*n*_/*γ*-Al_2_O_3_(110) suggests that the pre-adsorbed ethylene facilitated the nucleation from the even-sized supported Ir_*n*_ to the odd-sized Ir_*n*_ clusters, but hindered the nucleation from the odd-sized Ir_*n*_ to the even-sized Ir_*n*_ clusters. For ethylene pre-adsorbed Ir_*n*_ in the gas phase via the π mode, the pre-adsorbed ethylene hindered the nucleation of Ir_*n*_ (n = 2, 5, and 6), facilitated the nucleation of Ir_4_ from the Ir_3_ cluster, and had no effect on the nucleation of Ir_3_ from Ir_2_. 

## 4. Conclusions

Understanding the adsorption properties of ethylene on supported metal clusters at the atomic level is of great significance for the design of nanocatalysts and their applications in fine chemistry and petroleum refining. The interaction of ethylene with the bare and hydrated γ-Al_2_O_3_ (110)-supported Ir_*n*_ (*n* = 1–10) clusters was systematically studied by DFT calculations using periodic models. We first identified the most favorable configurations of Ir_*n*_ (*n* = 1–10) clusters on the γ-alumina support. For Ir_*n*_ (*n* = 1–6 and 8) with a cluster diameter smaller than 4.11 Å, the Ir_*n*_ cluster adsorbed on the surface O and Al sites only, while for Ir_*n*_ (*n* = 7, 9, and 10) with a cluster diameter larger than 4.21 Å, besides binding to the surface O and Al atoms, the Ir_*n*_ cluster binded to the oxygen of surface hydroxyl as well. The γ–Al_2_O_3_ (110) support changed the morphological features and the cluster stability of Ir_3_ and Ir_4_ upon their adsorption on the support.

The stability of ethylene adsorption on the bare Ir_*n*_ clusters decreased in the sequence of π > di-σ > B-T, with an exception of Ir_8_ where a preference of the di-σ structure over the π structure was found. Compared to ethylene adsorption on the bare Ir_*n*_ clusters, the *γ*-Al_2_O_3_ support reversed the stability of π and di-σ modes on the supported Ir_*n*_ (*n* = 2, 3, and 9) but kept the same for the other bare and supported Ir_*n*_ (*n* = 4–8 and 10) clusters. For supported Ir_*n*_ (*n* = 2, 3, 8, and 9), the stability of the ethylene adsorption mode decreased in the order di-σ > π > di-σ′ (at interface) while, on the supported Ir_*n*_ (*n* = 4–7 and 10), the sequence changed to π > di-σ > M > di-σ′ (at interface). M mode was only available on the supported Ir_4_ and Ir_6oct_ clusters. The carbon centers of the adsorbed ethylene completely rehybridized at the interface from *sp^2^* in the gas phase to *sp^3^* in the adsorbed state, while for adsorptions on Ir_*n*_, the orbital hybridization of the carbon centers in adsorbed ethylene was between *sp^2^* and *sp^3^*. 

Among 21 pairs (for example, the π mode on the bare Ir_1_ and supported Ir_1_ clusters counts as 1 pair), 6 ethylene adsorption modes on the supported Ir_*n*_ clusters were stronger than on the bare ones, including π and di-σ on Ir_2_, di-σ mode on Ir_3_, π and di-σ on Ir_6oct_, and π mode on Ir_7_. One pair—π mode on the supported and bare Ir_1_—showed similar stability. For the remaining 14 pairs, ethylene adsorption on the supported Ir_*n*_ clusters was weaker than on the bare ones.

Thermodynamic analysis showed that ethylene adsorption on the bare and supported Ir_*n*_ clusters was much more favorable than on the Ir(111) surface. The interface between the Ir_*n*_ clusters and *γ*-Al_2_O_3_ support provided a new adsorption mode di-σ′ (at interface), which was the weakest among all adsorption modes. 

The pre-adsorbed ethylene at the interface was found to facilitate the nucleation from the even-sized supported Ir_*n*_ to odd-sized Ir_*n*_ clusters, but hindered the nucleation from the odd-sized Ir_*n*_ to even-sized Ir_*n*_ clusters. 

The electronic analysis shows that the adsorbed ethylene induced charge redistribution between the support and metal clusters. The *d* band states became a little smoother for the supported Ir monomer, but still localized to the metal cluster upon ethylene adsorption, while for the supported Ir_10_ cluster, the *d* band states became sharper after ethylene adsorption. 

## Figures and Tables

**Table 1:** Adsorption energy E_ads_ (eV), standard adsorption Gibbs free energy at 300 K with the partial pressure of ethylene at 1atm ∆G^ɵ^_ads_(300 K) (eV), ethylene deformation energy E_def_(C_2_H_4_) (eV), Ir_*n*_ cluster deformation energy E_def_(Ir_*n*_) (eV), interaction energy E_int_ (eV), carbon–carbon bond distance d_C-C_ (Å), and mean hybridization value (hyd.) for ethylene adsorption on bare Ir_*n*_ (n = 1–10) clusters.

n	mode	E_ads_	∆G^ɵ^_ads_(300 K)	E_def_(C_2_H_4_)	E_def_(Ir_*n*_)	E_int_	d_C-C_	hyb.
1	π	−2.96	−3.70	0.63	0	−3.59	1.44	2.50
2	π	−1.79	−2.53	0.52	0.02	−2.33	1.44	2.44
	di-σ	−1.75	−2.49	1.85	0.15	−3.75	1.53	2.89
3	π	−1.82	−2.56	0.52	0.23	−2.57	1.43	2.45
	di-σ	−1.75	−2.49	1.53	0.15	−3.43	1.51	2.84
4squ	π	−2.46	−3.20	0.48	0.02	−2.96	1.43	2.42
	di-σ	−1.89	−2.63	1.70	0.11	−3.70	1.52	2.84
4ben	π	−2.25	−2.99	0.50	0.12	−2.87	1.43	2.42
	di-σ	−2.01	−2.75	1.60	0.11	−3.72	1.51	2.84
5	π	−2.30	−3.04	0.52	0.02	−2.84	1.43	2.44
	di-σ	−1.89	−2.63	1.71	0.04	−3.64	1.51	2.89
6tri	π	−2.06	−2.80	0.55	0.04	−2.65	1.44	2.45
	di-σ	−1.84	−2.58	1.65	0.01	−3.50	1.51	2.84
6oct	π	−2.02	−2.76	0.57	0.06	−2.65	1.44	2.47
	di-σ	−1.56	−2.30	1.75	0.14	−3.45	1.52	2.86
7	π	−1.94	−2.68	0.54	0.01	−2.49	1.44	2.42
	di-σ	−1.85	−2.59	1.68	0.17	−3.70	1.51	2.86
8	π	−1.63	−2.37	0.54	0.17	−2.34	1.44	2.46
	di-σ	−1.76	−2.50	1.74	0.08	−3.58	1.52	2.84
9	π	−2.11	−2.85	0.52	0.05	−2.68	1.44	2.44
	di-σ	−1.82	−2.56	1.72	0.02	−3.56	1.52	2.85
10	π	−1.88	−2.62	0.45	0.15	−2.48	1.43	2.43
	di-σ	−1.77	−2.51	1.71	0.13	−3.61	1.52	2.85

**Table 2:** Adsorption energy E_ads_ (eV), standard adsorption Gibbs free energy at 300 K with the partial pressure of ethylene at 1atm ∆G^ɵ^_ads_(300 K) (eV), ethylene deformation energy E_def_(C_2_H_4_) (eV), substrate deformation energy E_def_(Ir_*n*_/*γ*-Al_2_O_3_) (eV), interaction energy E_int_ (eV), carbon–carbon bond distance d_C-C_ (Å), and mean hybridization value (hyd.) for ethylene adsorption on hydrated *γ*-Al_2_O_3_(110)-supported Ir_*n*_ (n = 1–10) clusters.

n	mode^a^	E_ads_	∆G^ɵ^_ads_(300 K)	E_def_(C_2_H_4_)	E_def_(Ir_*n*_/*γ*-Al_2_O_3_)	E_int_	d_C-C_	hyb.
1	π	−2.95	−3.69	0.61	0.04	−3.60	1.44	2.51
	di-σ′	−0.53	−1.27	3.75	1.49	−5.77	1.52	3.00
2	π	−2.10	−2.84	0.53	0.19	−2.82	1.44	2.45
	di-σ	−2.26	−3.00	1.70	0.73	−4.69	1.51	2.80
	di-σ′	−0.48	−1.22	3.87	1.06	−5.41	1.51	2.98
3	π	−1.75	−2.49	0.55	0.11	−2.41	1.44	2.46
	di-σ	−2.15	−2.89	1.91	0.69	−4.75	1.52	2.93
	di-σ′	−0.58	−1.32	3.73	1.08	−5.39	1.51	2.96
4	π	−1.94	−2.68	0.48	0.32	−2.74	1.42	2.45
	di-σ	−1.79	−2.53	1.55	0.15	−3.49	1.51	2.84
	di-σ′	−0.44	−1.18	3.72	1.08	−5.24	1.48	2.97
5	π	−1.84	−2.58	0.52	0.02	−2.38	1.43	2.48
	di-σ	−1.59	−2.33	1.65	0.08	−3.32	1.51	2.84
	di-σ′	−0.64	−1.38	3.92	0.87	−5.43	1.51	2.96
6tri	π	−1.71	−2.45	0.48	0.20	−2.39	1.42	2.44
	di-σ	−1.54	−2.28	1.71	0.06	−3.31	1.52	2.87
	di-σ′	−0.56	−1.30	3.75	0.99	−5.30	1.51	2.96
6oct	π	−2.19	−2.93	0.58	0.24	−3.01	1.44	2.47
	di-σ	−1.91	−2.65	1.76	0.27	−3.94	1.52	2.86
	di-σ′	+0.82	0.08	3.69	1.58	−4.45	1.51	2.95
7	π	−1.96	−2.70	0.57	0.19	−2.72	1.44	2.48
	di-σ	−1.79	−2.53	1.42	0.48	−3.69	1.49	2.84
	di-σ′	−0.56	−1.30	3.72	0.85	−5.13	1.52	2.99
8	π	−1.52	−2.26	0.55	0.09	−2.16	1.44	2.46
	di-σ	−1.67	−2.41	1.75	0.07	−3.49	1.52	2.85
	di-σ′	−0.25	−0.99	3.68	0.90	−4.83	1.51	2.96
9	π	−1.61	−2.35	0.54	0.19	−2.34	1.43	2.49
	di-σ	−1.74	−2.48	1.49	0.38	−3.61	1.49	2.78
	di-σ′	−0.02	−0.76	3.67	1.09	−4.78	1.51	2.99
10	π	−1.59	−2.33	0.53	0.18	−2.30	1.43	2.48
	di-σ	−1.32	−2.06	1.57	0.13	−3.02	1.50	2.82
	di-σ′	+0.07	−0.67	3.66	1.10	−4.69	1.51	3.00

^1^ π and di-σ: two C atoms bond to Ir atom(s); di-σ′: di-σ mode at the interface where one C atom binds to O of the support and the other C atom binds to Ir.

## Supplementary Materials

The following are available online at https://www.mdpi.com/2079-4991/9/3/331/s1, Figure S1: M configuration of ethylene adsorption. Figure S2: Linear fitting of ethylene deformation energy E_def_(C_2_H_4_) (eV) and mean hybridization value of the carbon center in adsorbed ethylene. Table S1: Geometry, magnetic moment (*M*), and energy of gas phase Ir_*n*_ (n = 2–10) Table S2: Adsorption energy E_ads_ (eV) and Nucleation energy E_nuc_ (eV) for Ir_*n*_ clusters on *γ*-Al_2_O_3_ surfaces.
