# Supplementary material for: Interaction of Ethylene with Irn (n = 1–10): From Bare Clusters to γ-Al2O3-Supported Nanoparticles
Source: Nanomaterials (Basel). 2019 Mar 2;9(3):331. doi: 10.3390/nano9030331 (PMC6473975; doi:10.3390/nano9030331)
Supplement: Supplementary file 1 [file nanomaterials-09-00331-s001.pdf]

Supplemental files

Interaction of ethylene with Ir_n (n = 1-10): From bare clusters to γ-Al_2O_3-supported nanoparticles

Xue-Rong Shi [1,2]*, Yajing Zhang [1], Shibiao Zong [1], Wen Gu [1], Pan Ma [1], and Na Lu [1]

[1] School of Material Engineering, Shanghai University of Engineering Science, 333 Longteng Road, Songjiang District, Shanghai 201620, P. R. China; shixuer05@mails.ucas.ac.cn(X.-R.S.), M050118113@sues.edu.cn(Y.Z.), M050117110@sues.edu.cn(S.Z.), guwen1-1@163.com(W.G.), mapan@sues.edu.cn(P.M.), 05160003@sues.edu.cn(N.L.)

[2] Institute of Physical Chemistry, University of Innsbruck, Innrain 80-82, Innsbruck A-6020, Austria

* Correspondence: shixuer05@mails.ucas.ac.cn; Tel.: +86-21-6779-1380

Table S1: Geometry, magnetic moment (M), and energy of gas phase Ir_n (n = 2-10) clusters.

n	Geometry	M [μ_B]	d(Ir-Ir) [Å]	CE[a] [eV/atom]	BE[b] [eV]
2	$D_{\infty h}$	4(4[c])	2.21(2.21[c])	-2.68(-2.61[c])	-5.36
3	$D_{\infty h}$	1(1[c])	2.18(2.18[c])	-3.36(-3.27[c])	-5.03
4	D_{4h}	4(4[c,d])	2.31(2.31[c])	-3.92(-3.85[c], 3.77[d])	-3.92
5	C_{4v}	5(5[c,e])	2.47(2.47[c])	-4.16(-4.08[c], 4.01[d])	-2.60
6	D_{3h}	6(6[c,e])	2.42(2.42[c])	-4.54(-4.45[c], 4.38[d])	-3.03
7	C_{2v}	11(11[c,d,e])	2.51(2.51[c])	-4.64(-4.56[c], 4.51[d])	-2.50
8	O_h	0(0[c,d,e])	2.37(2.37[c])	-5.08(-5.00[c], 4.90[d])	-3.39
9	C_s	3(3[c,e])	2.38(2.39[c])	-5.08(-5.00[c], 4.90[d])	-3.27
10	C_{2v}	4(4[d,e])	2.42(2.42[c])	-5.23(-5.15[c],5.02[d])	-3.08

[a] Cohesive energy = $(E[Ir_n] - nE[Ir])/n$

[b] Bond energy = nCE/m where m is the number of Ir-Ir bonds in the cluster

[c] Ref.[1], VASP code, PW91 functional, 380 eV;

[d] Ref.[2], VASP code, PW91 functional, 300 eV;

[e] Ref.[3], $Dmol^3$ code, BPW91 functional

Table S2: Adsorption energy E_{ads} (eV) and Nucleation energy E_{nuc} (eV) for Ir_n clusters on γ-Al_2O_3 surfaces.

	E_{ads}		E_{nuc}	
N	γ-Al_2O_3(110)	γ-Al_2O_3(001)[a]	γ-Al_2O_3(110)	γ-Al_2O_3(001)[a]
1	-2.58	--	--	--
2	-2.53	-1.03	-2.73	-2.77
3	-3.67	-1.17	-3.01	-2.75
4	-4.12	-2.65	-3.00	-4.31
5	-3.74	-2.18	-2.92	-4.04
6	-3.92	-0.81	-4.05	-3.19
7	-3.38	-1.82	-2.18	-4.44
8	-2.16	-1.96	-4.33	-6.48
9	-2.74	-1.64	-3.06	-3.33
10	-2.98	-1.78	-4.25	-4.71

[a] Ref.[1], VASP code, PW91 functional, cutoff energy 380 eV.

M configuration of ethylene adsorption

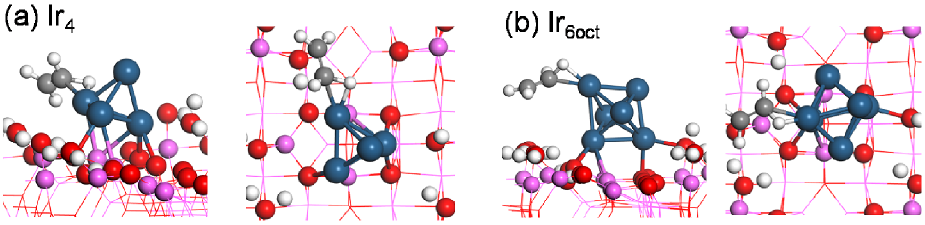

Figure S1: Most stable M configuration of ethylene adsorption on hydrated γ-Al_2O_3(110)-supported (a)Ir_4 and Ir_{6oct}.

Linear fitting of ethylene deformation energy $E_{def}(C_2H_4)$ (eV) and mean hybridization value of the carbon center in adsorbed ethylene.

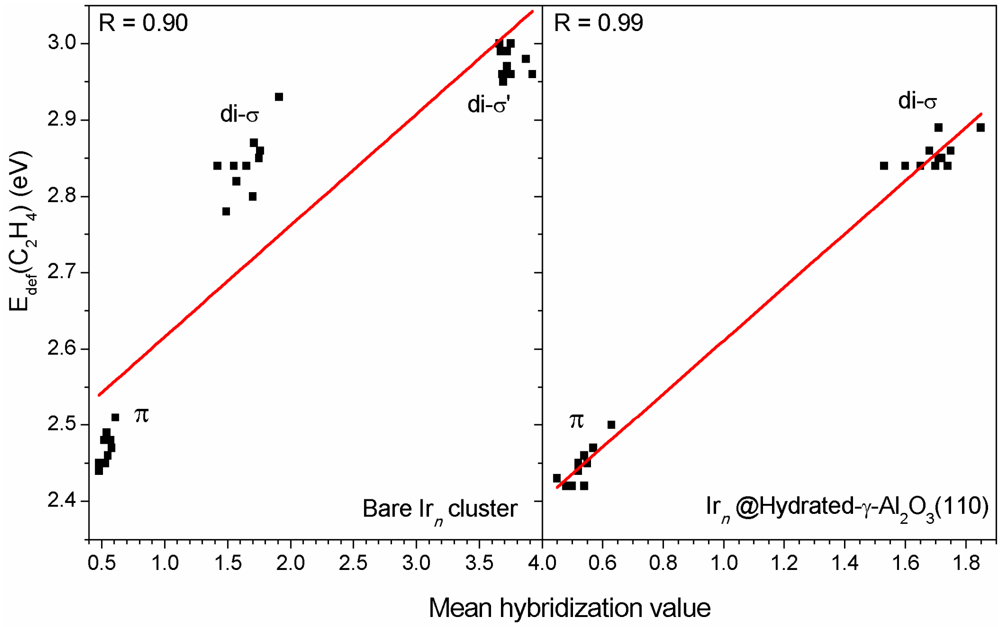

Figure S2: Ethylene deformation energy $E_{def}(C_2H_4)$ via different modes as a function of mean hybridization value of the carbon center in adsorbed ethylene. The straight lines refer to linear fits.

Reference

1. Chen, Y.; Huo, M.; Chen, T.; Li, Q.; Sun, Z.; Song, L. The properties of Ir_n (n = 2-10) clusters and their nucleation on γ-Al_2O_3 and MgO surfaces: from ab initio studies. *Phys Chem Chem Phys* **2015**, *17*, 1680–1687, doi:10.1039/c4cp04881d.

2. Pawluk, T.; Hirata, Y.; Wang, L. Studies of iridium nanoparticles using density functional theory calculations. *J Phys Chem B* **2005**, *109*, 20817–20823, doi:10.1021/jp053563b.

3. Du, J.; Sun, X.; Chen, J.; Jiang, G. A theoretical study on small iridium clusters: structural evolution, electronic and magnetic properties, and reactivity predictors. *J Phys Chem A* **2010**, *114*, 12825–12833, doi:10.1021/jp107366z.
